# Non-Coding RNAs in Tuberculosis Epidemiology: Platforms and Approaches for Investigating the Genome’s Dark Matter

**DOI:** 10.3390/ijms23084430

**Published:** 2022-04-17

**Authors:** Ahmad Almatroudi

**Affiliations:** Department of Medical Laboratories, College of Applied Medical Sciences, Qassim University, Buraydah 51452, Saudi Arabia; aamtrody@qu.edu.sa

**Keywords:** noncoding RNAs, tuberculosis, epidemiology, RNA informatics, RNA structure-function relationships

## Abstract

A growing amount of information about the different types, functions, and roles played by non-coding RNAs (ncRNAs) is becoming available, as more and more research is done. ncRNAs have been identified as potential therapeutic targets in the treatment of tuberculosis (TB), because they may be essential regulators of the gene network. ncRNA profiling and sequencing has recently revealed significant dysregulation in tuberculosis, primarily due to aberrant processes of ncRNA synthesis, including amplification, deletion, improper epigenetic regulation, or abnormal transcription. Despite the fact that ncRNAs may have a role in TB characteristics, the detailed mechanisms behind these occurrences are still unknown. The dark matter of the genome can only be explored through the development of cutting-edge bioinformatics and molecular technologies. In this review, ncRNAs’ synthesis and functions are discussed in detail, with an emphasis on the potential role of ncRNAs in tuberculosis. We also focus on current platforms, experimental strategies, and computational analyses to explore ncRNAs in TB. Finally, a viewpoint is presented on the key challenges and novel techniques for the future and for a wide-ranging therapeutic application of ncRNAs.

## 1. Background

Tuberculosis (TB) is an infectious respiratory disease, caused by an intracellular pathogen referred to as *Mycobacterium tuberculosis* (Mtb), which is most commonly transmitted via inhalation and spreads to the alveolar space [1]. Since ancient times, tuberculosis has been a lethal disease that has posed a threat to public health, resulting in mortality [2]. A pandemic of tuberculosis in 1993 led the World Health Organization [3] to declare TB a global health emergency [3]. In accordance with the WHO, approximately ten million individuals were infected with TB, and approximately 6.4 million were newly diagnosed [4]. Furthermore, it was stated that about one million die from the disease every year [5].

As drug-resistant strains of Mtb have emerged, tuberculosis treatment has become more challenging [6]. Regardless of the fact that most of the research on tuberculosis has paid attention to the protein coding genes, the fact that approximately 97 percent of the human genetic material is composed of non-coding segments has prompted scientists to explore the genetic dark matter of tuberculosis [7]. The term untranslated RNAs refers to non-coding RNAs (ncRNAs) [8]. Micro-RNAs (miRNAs), which are short ncRNAs 22–25 nt; long non-coding RNAs (lncRNAs), which are the largest class of ncRNAs, with approximately 55,000 genes in human genetic material [7]; and circular RNAs (circRNAs) are the most extensively studied [9]. As ncRNAs exhibit tissue-specific expression patterns that are significantly dysregulated in tuberculosis, they are attractive diagnostic, prognostic, and therapeutic targets. As a result, elucidating the role of noncoding RNAs in tuberculosis in modern biology is a difficult task [10]. In the current review paper, we explain in silico and in vitro approaches to explore the noncoding RNA transcriptome, with a complete overview of strategies and tools for characterizing ncRNA structure and examining their role in tuberculosis and progression. Additionally, we discuss the implications of these approaches for translation.

## 2. Classification and Biogenesis of Noncoding RNAs

Only about 20,000 protein-coding genes are found in human DNA, accounting for less than 2% of the total genetic material. It was discovered using cutting-edge technologies that approximately 90% of the genome is actively transcribed [11]. The transcriptome of humans contains antisense, overlapping, and noncoding RNA expression, which makes it more complex than a collection of coding genes and splice variants [12]. Around 97 percent of the human genome is non-coding, prompting scientists to explore this genetic dark matter in a variety of diseases, including tuberculosis [13]. Certain classes of noncoding RNAs, called housekeeping ncRNAs, are expressed ubiquitously in all types of cells [14]. Transfer RNAs (tRNAs) and ribosomal RNAs (rRNAs) are two distinct types of RNAs involved in messenger RNA (mRNA) [15].

**Figure 1:**
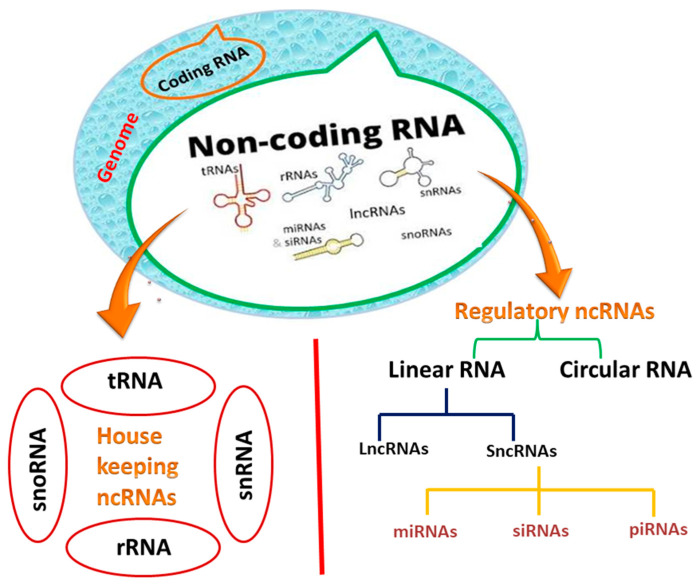
Classification of non-coding RNAs (ncRNAs).

Another family of noncoding RNAs has recently sparked interest in the scientific community because of its regulatory roles [8]. Regulatory noncoding RNAs are classified into two types: (i) linear RNAs containing sncRNAs (small non-coding RNAs), which are further subdivided into microRNAs (miRNAs), small interfering RNAs (siRNAs), and long non-coding RNAs (lncRNAs); (ii) and circular RNAs (circRNAs) (Figure 1) [16]. Unlike linear RNAs, circRNAs can make a covalently closed loop in the absence of 5′ and 3′ polar regions. In the current review, we will concentrate on miRNAs and lncRNAs, including circular RNAs, and their biogenesis and roles in tuberculosis [17].

### 2.1. miRNA

**Figure 2:**
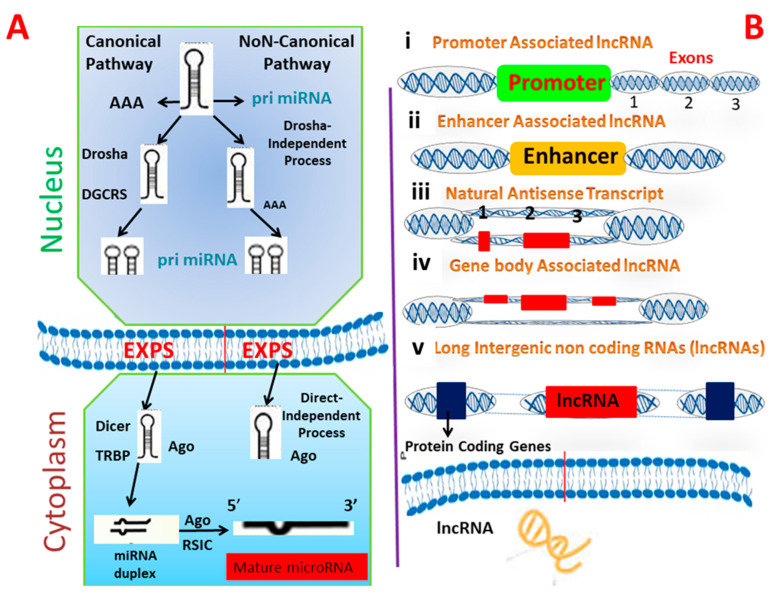
Schematic representation of biogenesis pathway of microRNA (**A**) and lncRNA (**B**). Number (i,ii,iii,iv) in (**B**) shows the number of biogenesis steps of lncRNAs while (1,2,3) shows the number of exons.

miRNAs are a subclass of noncoding short RNAs that range from 18 to 22 nucleotides (nt) [18]. MicroRNAs are crucial posttranscriptional regulators of gene expression and their roles in the pathogenesis of infectious diseases are well understood; they may be used as diagnostics. miRNAs are thought to be more stable biomarkers than mRNA, because of their small size and chemical structure [19]. Indeed, numerous recent publications suggest that changes in host’s miRNA expression are a common sign of bacterial infection at the cellular and organismal levels in humans, including those caused by Mtb [20]. All miRNAs undergo multistep maturation and processing steps that start in the nucleus and end in cytoplasm. RNA polymerase II synthesizes primary miRNA transcripts, in the form of a massive double-stranded primary transcript known as pri-miRNAs, which can be some 100 nt in length [21] and available in polyadenylated and capped hairpin forms. Drosha, an RNase III-like enzyme, converts this precursor into a double stranded miRNA precursor composed of 60–100 nt hairpins, referred to as pre-miRNAs. Pre-miRNAs have a stem-loop structure and are traded from nucleus to cytoplasm by exportin 5 [22]. Further processing of pre-miRNAs in the cytoplasm through RNase III Dicer enzyme results in the generation of an incomplete 22 nt double-stranded miRNA. The guide strand (miRNA) and the passenger strand (miRNA*) combine to form this unstable duplex [23]. The miRNA* is eliminated, while the guide strand miRNA matures. After maturation, mature miRNAs are integrated into the RNA-induced silencing complex (RISC), which distinguishes particular targets and promotes posttranscriptional gene silencing; thereby regulating gene expression. Recently, it was discovered that a novel biogenesis pathway exists in which microRNA enters RISC without being further processed by Dicer [24]. The strand enters RISC following Drosha processing (Figure 2A). The role of microRNA is well-defined by the genes that it targets and the effect of it on their expression. MiRNAs interact with bacteria in a variety of ways, depending on the bacteria’s characteristics [25].

By regulating apoptosis in TB-infected cells and utilizing the host’s energy and metabolic processes, miRNAs contribute significantly to the pathogenesis of Mtb infection [26]. Hence, when Mtb is in the host, it changes the expression of miRNA genes.

Remarkably, anti-TB treatment restored the previously reduced expression of numerous miRNAs [27]. These findings imply that miRNAs provide the induction of inflammatory responses in human cells in response to bacterial pathogens. Future research on the miRNAs’ role and expression in tuberculosis may advance our understanding of Mtb pathogenesis and point the way toward improved diagnostics and/or novel treatment options [28].

### 2.2. lncRNAs

lncRNAs are a group of RNAs that do not undergo translation and are more than 200 nt long. Instead, they function as an RNA molecule. As with other mRNAs, long non-coding RNAs have a distinct 5′ cap structure and a 3′ polyadenylate structure, and were previously thought to lack an open reading frame [29]. Additionally, these RNAs are categorized according to their position relative to the coding gene, with the most common types of lncRNA such as forward long ncRNA, reverse lncRNAs, intragenic, bidirectional, and intergenic lncRNAs [30]. The presence of lncRNAs encoded short functional peptides shows that they can function simultaneously in RNA, as well as in peptides. Recognizing the biogenesis of lncRNAs is critical for differentiating them from the other types of RNA and elucidating their role and characteristics [31]. lncRNAs biogenesis is stage- and cell-type-specific, and is regulated by cell- and stage-specific stimuli. Numerous long noncoding RNAs (lncRNAs) are transcribed from diverse DNA genomic components, including promoters, and intergenic regions. Numerous mechanisms for the formation of lncRNAs have been proposed, including ribonuclease P cleavage of mature ends, the formation of small nucleolar protein (snoRNP) and snoRNA complex caps in ends and the formation of circular structures [32]. Additionally, distinct subnuclear structures known as paraspeckles were discovered recently surrounding specific lncRNAs during biogenesis (Figure 2B). The term “paraspeckles” refers to nuclear particles that are frequently observed near the nucleus in small foci. Paraspeckles may contribute to lncRNA-dependent gene expression, by transporting messenger RNA into the nucleoplasm in response to particular stimuli [33]. While the mechanisms underlying the biogenesis of various lncRNAs are unknown at the moment, additional information is expected to be obtained in the coming years as a result of the development of techniques such as RNA structure mapping, chromatin isolation by ribosome profiling, phylogenetic lineage tracing, RNA purification (ChIRP-Seq), and crosslinking immunoprecipitation (CLIP) [34,35]. Numerous long noncoding RNAs play a role in the regulation of biological processes such as cell lineage determination, cell organogenesis and differentiation, and tissue homeostasis in a wide range of diseases, including rheumatoid arthritis, tuberculosis, diabetes, and various types of cancer [36]. Most of the lncRNAs that are linked to diseases have a dysregulated expression in different cells. Dysregulation of long ncRNAs has been found in many infections, such as *Mycobacterium tuberculosis*, *Salmonella typhimurium,* and *Escherichia coli* [37].

### 2.3. CircRNAs

**Figure 3:**
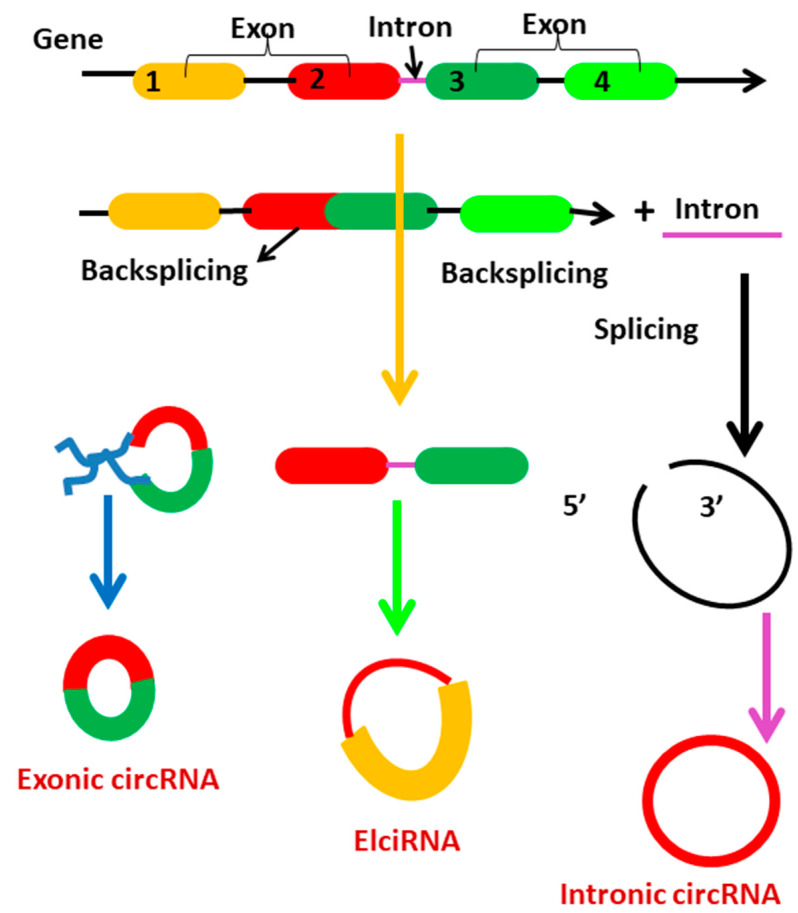
Representation of circRNA biogenesis. Numbers (1,2,3,4) in fig represents the exon 1, exon 2, exon 3 and exon 4.

Circular RNAs are generated from primary transcripts via back-splicing and lariat-driven circularization, which compete with mRNA splicing [38]. Circularization of backsplicing occurs in response to cis- and trans-acting components and variables. Circularization occurs when cis-acting elements in upstream introns are base-paired with those in downstream introns [39]. TAFs are RNA-binding proteins (RBPs) that promote circularization through interacting with particular motifs within flanking introns. Circularization-driven circularization is another possibility for the biogenesis of circRNAs [40]. This occurs when pre-mRNA exons or introns are skipped or removed. Circular RNAs are classified according to their biogenesis into three types: exonic circRNAs (EcRNAs), exon-intron circRNAs (EIciRNAs), and circular intronic circRNAs (ciRNAs) (Figure 3). While both EIciRNAs and ciRNAs are circular RNAs with nuclear introns, most EcRNAs are synthesized in cytoplasm. While the majority of circular RNAs are synthesized from precursor mRNA, a minority are synthesized from precursor tRNA [41]. During tRNA maturation, the tRNA splicing endonuclease (TSEN) complex may cleave intron-containing pre-tRNA. RtcB ligase cyclizes intron termini, forming circular tRNA intronic RNAs (tricRNAs) [42].

## 3. Role of ncRNAs in the Molecular Epidemiologic Studies of Tuberculosis

### 3.1. The Role of miRNA

miRNA regulatory dysfunction is required for biological processes [43] and has been associated with a number of human diseases, including tuberculosis. Specifically, miRNA dysregulation could help us better understand human diseases [44]. Anomalies manifest in a variety of ways: (i) down regulation of microRNA expression due to mutation, or transcriptional downregulation; (ii) overexpression of microRNA due to gene amplification or transcriptional upregulation, which could result in decreased production of its target proteins; (iii) mutation in the 3UTR of mRNA can affect a microRNA binding site, rendering the microRNA inactive; and (iv) mutation in the miRNA itself may cause decreased production of its target proteins [45,46]. Numerous studies have established a link between miRNA dysregulation and tuberculosis. Recent research has established that the control of miRNA in tuberculosis can only be described by genetic or epigenetic abnormalities in the processing system [47]. This insight has been applied to a variety of infectious diseases, implying that miRNA could be used as clinical biomarkers [48]. Many studies have found that macrophages and natural killer cells from tuberculosis patients have different gene expression profiles when they are active and when latent [49].

This alteration in tuberculosis patients’ cellular makeup and gene expression is almost certainly caused by miRNAs. Numerous microRNAs have been identified to play a role in the regulation of T cell development and function. Additionally, microRNAs are involved in the regulation of macrophages and natural killer cells’ innate functions [50]. Next, we discuss the mechanism of action of a few miRNAs in the context of metabolic pathways.

#### 3.1.1. miRNA-29

In vitro and clinical investigations have demonstrated that miR-29 is overexpressed in various human cell types, following infection with pathogenic Mycobacterium species [51]. By downregulating IFN-g, miR-29 reduces immunological responses to Mycobacterium TB [52]. Apart from targeting IFN-g mRNA at the 30UTR, miR-29a stimulates the interaction of IFN-g mRNA with Argonaute 2 (Ago2) protein, resulting in the formation of an RNA-induced silencing complex and subsequent suppression of IFN-g expression post-transcriptionally [53]. Additionally, several reports have indicated that miR-29 targets the antiapoptotic proteins B-cell lymphoma 2 (Bcl-2) and myeloid cell leukemia-1 (Mcl-1), as well as the kinase p85a and the GTP-binding protein Cdc42; implying that miR-29 plays a critical role in regulating the apoptotic pathway in immune cells [54]. Thus, overexpression of miR-29 in TB infection contributes one method by which *Mycobacterium tuberculosis* avoids macrophage digestion, by inhibiting IFN-g and boosting apoptosis of cells engaged in anti-tuberculosis responses [55]. Intriguingly, a contrary occurrence happened in a model of non-virulent Mycobacterium species infection [56]. In NK cells and T cells, Mycobacterium bovis Bacillus Calmette-Guerin (BCG) inhibited miR-29 expression and elevated IFN-g expression [57]. This result suggests that silencing miR-29 may have promoted the generation of IFN-g by these T cells and that miR-29 expression is controlled by Mycobacterium species-specific pathogenicity.

#### 3.1.2. miRNA-147

A previous study demonstrated that miR-147 is increased in macrophages via the TLRs/NF-kB signaling pathway and inhibits the expression of proinflammatory cytokines such as TNF-a and IL-6 [58]. These findings suggest that miR-147 possesses significant anti-inflammatory activity. TNF-a and IL-6 levels in serum or peripheral blood mononuclear cells (PBMCs) were shown to be significantly higher in active tuberculosis compared to healthy controls [59]. A pivotal study discovered that miR-147 was significantly overexpressed in sputum taken from patients with active tuberculosis, as compared to controls [60]. Interestingly, this study discovered that the levels of TNF-a and IL-6 in sputum were not significantly different between active tuberculosis patients and controls, indicating that dysregulation of cytokines occurs mostly in the circulation, rather than the lungs [61]. Taken together, these findings suggest that miR-147 and miR-29 augment anti-tuberculosis immunity and operate as a negative regulator of the immune response to tuberculosis.

#### 3.1.3. miRNA-21

Previous research indicates that miR-21 inhibits the expression of proinflammatory cytokines and promotes the synthesis of an anti-inflammatory cytokine, IL-10 [62]. A recent study discovered that miR-21 is increased in unsensitized DCs and macrophages following M. bovis BCG infection, both in vitro and in vivo, via the TLR/Erk/NF-kB pathway [63]. miR-21 expression is also increased when macrophages are challenged with the Mycobacterium TB early-secreted antigenic target 6 kDa protein (ESAT-6) antigen [64]. By directly targeting the 30UTR of IL-12 mRNA, miR-21 decreased host Th1 responses. Additionally, this study discovered that miR-21 decreased the expression of protective cytokines (TNF-a and IL-6) in *M. tuberculosis* infection, although these cytokines’ levels were not significantly altered in response to ESAT-6 exposure. Notably, miR-21 increased DC death by targeting Bcl-2; corroborating prior findings that *M. tuberculosis* can induce apoptosis in infected cells. However, the precise mechanism by which miR-21 influenced Bcl-2 expression remains unknown. Additionally, miR-21 inhibitors increased IL-12 production and elicited more robust antimycobacterial responses [65,66]. As a result, miR-21 may be a useful method for Mycobacteria to circumvent the host immune response and establish chronic infection.

#### 3.1.4. miRNA-99b

Exposure to Mycobacterium TB but not to LPS increased miR-99b expression in murine DCs from MyD88-deficient mice. Further investigation revealed that inhibiting miR-99b (through antagomirs and knockdown) dramatically decreased *M. tuberculosis* growth and considerably increased proinflammatory cytokines such as TNF-a, IL-6, IL-12, and IL-1b. *M. tuberculosis* growth inhibition could be a result of an increase in the production of these proinflammatory cytokines [67,68,69]. Additionally, this study discovered that miR-99b directly targeted the tumor necrosis factor receptor superfamily, member 4 (TNFRSF-4) and TNF-a messenger RNA, to regulate the expression of a variety of cytokines and transcription factors involved in T cell differentiation pathways and tuberculosis clearance [70]. Additionally, treatment of DCs transfected with anti-miR-99b antibody resulted in an increase in bacterial mortality [71]. These findings demonstrate that miR-99b plays a critical role in *M. tuberculosis* development in DCs, by suppressing the production of TNF-a, which enables the bacteria to evade host protective immunological responses and survive within host phagocytes.

#### 3.1.5. miRNA-125b

Rajaram et al. discovered that incubating human macrophages with Mycobacterium TB and its component, lipomannan, increases miR-125b expression, while decreasing TNF production [72,73]. miR-125b directly targets the 30UTR of the TNF mRNA transcript, inhibiting translation and, perhaps, accelerating its degradation; hence, inhibiting TNF production. Additionally, this study discovered that only virulent Mycobacterium species inhibit the activation of the mitogen-activated protein kinase (MAPK) p38 and Akt, two components that significantly contribute to TNF generation in mycobacterial-infected macrophages [74,75]. Additionally, miR-125b increases the stability of kB-Ras2, an NF-kB signaling inhibitor in human macrophages, thereby suppressing the inflammatory response. Taken together, these findings indicate that *M. tuberculosis* inhibits TNF production by increasing miR-125b expression. By contrast, infection with avirulent Mycobacterium species and exposure to lipomannan decreased miR-125b expression, which was associated with increased TNF production [76]. Further investigation revealed that silencing miR-125b increased the stability of TNF mRNA and promoted proinflammatory responses.

#### 3.1.6. miRNA-155

miR-155 was discovered to be a multifunctional miRNA involved in a variety of biological processes, including infection, inflammation, and immunology. Previous research demonstrated that when miR-155 was knocked down in mice, more IL-4 and less IFN-g were produced, implying that miR-155 plays a critical role in regulating T cell-dependent responses. One study discovered contradictory data about miR-155 dysregulation in human macrophages infected with several Mycobacterium species [77,78]. The study discovered that *M. tuberculosis* infection and lipomannan exposure to human macrophages downregulated miR-155, resulting in decreased TNF production. This inhibition was mediated via the TLR-MAPK/Akt pathway. Further investigation demonstrated that *M. tuberculosis* inhibits the start and stability of TNF mRNA, resulting in decreased translation [79,80]. These findings, however, contradict a prior study, in which it was discovered that miR-155 targeted the 30UTR of the inositol phosphatase SH2-containing inositol 5-phosphatase (SHIP1) mRNA, a negative regulator of TNF production, resulting in its degradation and, thus, enhanced TNF production [81]. The other study discovered that overexpression of miR-155 increased TNF production by increasing the stability and half-life of mRNA.

#### 3.1.7. miRNA-144*

miR-144* (present on complementary passenger strand) is overexpressed in patients with active TB. miR-144* targets genes involved in the Janus kinase/signal transducers and activators of transcription (Jak-STAT) signaling circuit, the MAPK signaling pathway, the Toll-like receptor signaling pathway, and interactions with cytokine receptors [82,83]. Further transfection of T cells with the miR-144* precursor revealed that miR-144* inhibits the production of TNF-a and IFN-g [84]. Given the critical function of TNF-a and IFN-g in protective immunity, it is possible that miR-144* has an effect on the development and outcome of tuberculosis. miR-144* may influence anti-tuberculosis immunity by modifying cytokine production and T cell proliferation.

#### 3.1.8. miRNAs-223 and 424

Wang et al. [85] discovered that miR-223 and miR-424 were substantially expressed in the peripheral blood mononuclear cells (PBMCs) of patients with active tuberculosis [86,87]. A miR-223 silencing study in mice revealed an increased proportion of granulocytes, which are morphologically mature, hypersensitive to activating stimuli, and possess more fungicidal activity [87]. miR-424 stimulates monocyte development and then inhibits the transcription factor NFI-A78 expression. Wang et al. [85] discovered that several miRNAs target basic leucine zipper transcription factor 2/BTB and CNC homology 1 (BACH2) and B-cell CLL/lymphoma 7A (BCL7A) in active tuberculosis [88]. Wang et al. hypothesized that the decreased expression of these two genes, which are regulated by multiple miRNAs, such as miR-223 and miR-424, may result in an imbalance in the proportions of T cells and B cells in active tuberculosis patients, disrupting the delicate balance of immune control during *Mycobacterium tuberculosis* infection. Wang et al. [88] also discovered that miR-424 regulates the differentiation of B cells from plasma cells by targeting BACH2, BCL2, and BCL7A, which are important in cellular differentiation and development.

### 3.2. The Role of lncRNA

lncRNAs primarily regulate gene expression via chromatin remodeling, RNA–RNA interaction, transcriptional and translational activation or inhibition, and miRNA regulatory modulation [89]. mRNA splicing is one of the processes that lncRNAs can affect. For instance, it is unknown how the lncRNA MALAT1 regulates Ser/Arg splicing factors [90]. LncRNAs can affect the stability and trafficking of messenger RNA and proteins in the cytoplasm. Additionally, certain long noncoding RNAs are like a molecular sink or sponge for the proteins that interact with RNA (RBPs) [91]. RBPs function as chromatin modifiers, regulatory proteins for adjacent genes encoding proteins and transcription factors. MALAT1, snoRNAs, and NEAT1 are all instances of long ncRNAs that function as miRNA sponges, inhibiting/activating gene expression [92]. Certain lncRNAs can be synthesized from enhancer elements, which are frequently activated in a similar fashion to enhancers. Enhancer elements are DNA segments that have been covalently linked to specific proteins and function as transcriptional activators and enhancers for their target genes. Numerous enhancer regions produce RNAs called eRNAs or elncRNAs, which stand for enhancer-associated long noncoding RNAs [93,94]. Recent findings indicated that elncRNAs promote gene expression by increasing the accessibility of chromatin. Numerous functional lncRNAs, as mounting evidence indicates, exhibit variable expression patterns during microbial infections. These long noncoding RNAs have an essential function in regulating the interaction of host and pathogen. They originate in the host or are encoded by pathogen agents [95]. They can be activated as a result of pathogen growth and proliferation regulation or as a result of antimicrobial defense mechanisms. Here, we discuss the mechanism of action of a few lncRNAs in the context of their pathways.

#### 3.2.1. lncRNA MEG3

Downregulation of lncRNA MEG3 in TB led in an increase in cell proliferation, prevention of apoptosis, and stimulation of autophagy [96]. To address this issue, a study using a variety of approaches to determine the involvement of lncRNA MEG3 in infected macrophage autophagy discovered that silencing lncRNA MEG3 expression increases autophagy in mycobacteria-infected macrophages [97]. On the other hand, interferon-induced autophagy in infected macrophages was associated with the persistence of MEG3 downregulation for more than 24 h.

#### 3.2.2. lncRNA CD244

lncRNA CD244 and its signaling-related molecules are upregulated in tuberculosis infection, inhibiting T cell-dependent immunological responses and decreasing TNF- and IFN- production. Excessive production of lncRNA-BC050410, also known as lncRNA-CD244, has been shown to alter CD244 signaling and expression in the CD8+CD244+ T-cell subset [98]. CD244 lncRNA controls methylation events at the tnfa/infg loci and decreases the production of TNF-/IFN- in CD8+ T cells [99]. This inhibitory effect can be reversed by silencing lncRNA-CD244. Transfer of lncRNACD244–repressed CD8+ T cells reduced tuberculosis infection and pathology in Mtb-infected mice when compared to a control group with lncRNACD244–expressed CD8+ T cells [100]. It has been hypothesized that lncRNA-CD244 may be a significant target for tuberculosis infection treatment methods. CD4+ T cells play a critical role in the host defense against Mycobacterium TB, by modulating immunological homeostasis and regulating intracellular pathogen development.

#### 3.2.3. lncRNA NEAT1

Recent research reveals that NEAT1 expression increases during Mtb infection and may be connected with TB prognosis. Reduced expression of NEAT1 may impair macrophage clearance of intracellular Mtb [101]. Additionally, TB infection dramatically boosted NEAT1 expression in T helper-1 cells. Using siRNA and NEAT1 knockout mice, we found that increased NEAT1 expression during tuberculosis infection was related with an increase in infection duration and that decreased NEAT1 expression may induce macrophage clearance of *Mycobacterium tuberculosis* from cells [102,103].

#### 3.2.4. lncRNA PCED1B-AS1

lncRNA PCED1B-AS1 is expressed at a lower level in CD14+ monocytes from active tuberculosis patients than in healthy persons [104]. According to Li et al. [105] downregulation of the long noncoding RNA PCED1B-AS1 may decrease apoptosis and promote autophagy in infected macrophages [106]. Indeed, PCED1BAS1 as an endogenous sponge reduces miR-155 expression in macrophages via direct binding to miR-155 and abolishes miR-155’s influence on the target gene FOXO3/Rheb [107]. Thus, Mtb-induced PCED1B-AS1 downregulation lowers FOXO3 and Rheb expression, while increasing LC-3 expression and simultaneously decreasing caspase-3 expression and attenuating apoptosis [100,108]. Given this, PCED1B-AS1 may be used to augment autophagy in macrophages as a potential early diagnostic biomarker for active tuberculosis.

### 3.3. The Role of circRNA

CircRNAs perform several important roles, including modifying the expression of regulating gene transcription, functioning as microRNA sponges, binding to protein [109]. While the bulk of circRNAs are traded to the cytoplasm, a subclass of intron-carrying circRNAs are carried to the nucleus. Both EIciRNAs and ciRNAs regulate hereditary genes in a cis-regulatory manner [110]. EIciRNAs connect with U1 snRNP to create the EIciRNAs–U1 snRNP complex, which functions in conjunction with polymerase II (Pol II) to control host gene transcription in the promoter region. Similarly, circRNAs are accumulated at transcriptional sites to promote Pol II elongation, resulting in cis-regulatory effects on hereditary genes [111].

MiRNA sponges are widely regarded as one of circRNAs’ most critical functions. microRNAs are believed to operate as transcriptional regulators, connecting with particular mRNA locations to govern both pathological and normal gene expression. Numerous circRNAs have been identified as miRNA sponges [112]. CircRNAs have been demonstrated to influence gene expression by acting as protein sponges/decoys. For instance, circRNA100146 was shown to bind numerous types of the splicing factor family (SF3), which are highly associated with transcriptional regulation of genes among the upper hundred proteins, including SF3A1, SF3B2, and SF3B3 [113]. CircRNAs also act as scaffolds for proteins, stimulating chemical reactions or inhibiting the function of proteins. As exemplified by circ-Foxo3, which may operate as a protein scaffold for MDM2 and p53; hence, promoting p53 degradation. Numerous circular RNAs regulate gene expression and have a quick response to external stimuli via their association with particular proteins [114]. We next discuss the role of few circRNA in the context of metabolic pathways.

#### 3.3.1. circRNA 051239

According to Liu et al. [105], circRNA 051239 was highly elevated in drug-resistant tuberculosis patients, suggesting that circRNA 051239 may operate as miR-320a sponges and play a critical role in the development of tuberculosis drug resistance [115]. The sponge of circRNA 051239 is involved in cytokine production regulation, and decreasing miR-320a expression may enhance tuberculosis progression, by reactivating cell migration and proliferation in lung tissue [116].

#### 3.3.2. hsa-circRNA-100237

Previous research indicates that hsa-circRNA-100237 may play a role in tuberculosis pathogenesis by altering macrophage activities [108]. Due to the fact that the level of hsa-circRNA-100237 decreased during ATBI, it is possible that downregulated hsa-circRNA-100237 acted as a miR-33 sponge, promoting lipid storage by reducing mitochondrial fatty acid oxidation [117].

#### 3.3.3. circAGFG1

circAGFG1 promotes autophagy and inhibits apoptosis in macrophages infected with Mycobacterium TB by targeting miRNA1257 to control Notch signaling. Additionally, the action of circAGFG1 may be mimicked in normal cells by suppressing miRNA-1257 [118]. Notch2 is a critical component of the Notch signaling pathway and was discovered to be increased in tuberculosis patients. Recent research suggests that by inhibiting the Notch signaling system, the Th1/Th2 imbalance in tuberculosis patients can be corrected [119].

#### 3.3.4. CircTRAPPC6B

By targeting microRNA-874-3p, CircTRAPPC6B inhibits intracellular *Mycobacterium tuberculosis* growth, while promoting autophagy in macrophages. circ TRAPPC6B is formed from the TRAPPC6B gene’s exons 3 and 4. RNAse R digestion is ineffective in reducing circTRAPPC6B synthesis via PCR, implying a circular shape for circTRAPPC6B [120]. Along with the low level of expression of circTRAPPC6B in tuberculosis patients, it was discovered that anti-tuberculosis medication can restore its expression.

## 4. ncRNAs as a Diagnostic and Therapeutic Tool for Tuberculosis

### 4.1. The Potential of miRNAs as Diagnostic and Therapeutic Biomarkers for Tuberculosis

The diagnosis of tuberculosis is complicated by the low specificity and sensitivity of the currently available diagnostic assays. TB must be identified early, in order to prevent infection transmission and control the disease [114]. Thus, it would be extremely beneficial to develop novel tuberculosis biomarkers. However, examining microRNA profiles in tuberculosis patients prior to, and following, treatment may be beneficial. There is indication that changes in miRNA profiles are associated with therapeutic response [121]. Thus, the microRNA profile in serum can be used as a biomarker for tuberculosis and multidrug-resistant tuberculosis diagnosis and monitoring (Table 1). For instance, serum miR-125a-5p levels are increased in well-treated tuberculosis patients, but miR-148 b-3p miR-21-5p, and miR-92a-3p, levels are decreased. Additionally, following a directly observed treatment short-course (DOTS), miR-155 and miR-29a expression was increased, whereas miR-326 expression was decreased, and their expression was associated with decreased Th1 responses [93]. Furthermore, when compared with treated-patients, new TB-treatment patients, and healthy participants, patients with multidrug-resistant tuberculosis (MDR)-TB had the lowest serum level of miR-16. Intriguingly, regulatory miRNAs can be packaged in exosomes and transported to other cells, where they make changes in the target cell’s transcriptome and function, thereby serving as tuberculosis biomarkers [122]. For instance, it was discovered that the levels of exosomal miR-96, miR-484, and miR-425 in serum of tuberculosis patients correlate with infection grade and serve as a marker of diagnostic power [123]. Thus, combining miRNA measurements with established diagnostic markers may aid in tuberculosis detection and surveillance. However, these procedures are not without risk. The primary difficulty in this situation is that the detected miRNAs lack repeatability and diagnostic specificity. Despite numerous studies, the reported miRNA signatures are inconsistent [124]. These discrepancies may be explained by the source of the samples, which may be serum or plasma. Numerous findings have established that blood microRNAs instigate from a variety of tissues in healthy and sick individuals and that microRNAs in venous and arterial plasma exhibit distinct profiles of expression from those found in tissues [125]. Additionally, variations in results can be attributed to the sampling procedure, including sample preservation and processing, necessitating protocol standardization. The cohort size of a biomarker study should be representative of the population, and, thus, large enough to distinguish between healthy and diseased states [126]. Given the well-established role of miRNAs in tuberculosis infection outcome, interest in tuberculosis-specific miRNA targeting has increased. Thus, miRNAs administered to the lung may alter the lung’s resistance to microbial infections, providing an intriguing new approach to tuberculosis management and therapy [127]. One possible treatment technique is the administration of miRNAs associated with tuberculosis infection, such as miR-20, miR-27a, miR-155, mir-146a, or miR-33/33*. miR-155 is a frequently reported miRNA. MiRNA plays a complex role in tuberculosis and its biology, involving numerous pathways in the control of tuberculosis infection [128]. For instance, in the presence of Mtb, this miRNA enhances the activity of natural killer cells (NK cells). MiR-155, on the other hand, inhibits autophagy in infected macrophages by targeting a brain-expressed Ras homolog (Rheb). MiR-33/33* silencing promotes autophagy activation [129]. miR-23a specifically targets TLR2/MyD88/NF-B pathway-linked genes that influence the induction of autophagy and Mtb survival, implying that it may be used as a tuberculosis therapeutic target. Additionally, miRNAs playing roles in innate immunity or miRNAs targeting genes linked with cytokines may be reviewed for miRNA-based therapeutics. For instance, miR-99b stops the release of pro-inflammatory cytokines during *Mycobacterium tuberculosis* infection, whereas miR-20b inhibited the tuberculosis-induced inflammatory response in vivo by the NLRP3/caspase-1/IL-1 pathway. Anti-miR-mediated silencing of miRNAs is now hailed as a potentially game-changing tool used to treat infectious diseases. For example, miravirsen is a licensed anti-miR-122 oligonucleotide that has been modified with locked nucleic acid [130,131]. However, before the optimal settings for future miRNA-based tuberculosis therapy can be determined, these novel methods of miRNA delivery must be validated. Recent technological advances in gene delivery may facilitate the development of novel host-directed therapies (HDT) that specifically target miRNAs [132].

### 4.2. LncRNAs as Diagnostic and Therapeutic Biomarkers for Tuberculosis

lncRNAs associated with infectious diseases are expressed abnormally in various tissues and cells [132]. Long ncRNAs are increasingly being implicated in regulatory processes and are being used as molecular markers for a variety of infections [133]. Disruption of long noncoding RNAs has been linked to many infections caused by *S. typhimurium*, *M. tuberculosis*, etc. A microarray analysis of plasma lncRNAs and mRNA expression levels in tuberculosis victim was performed in this regard. 348 long ncRNAs and 284 messenger RNAs were found to be downregulated, while 163 long non-coding RNAs were found to be upregulated [134]. The Kyoto encyclopedia of genes and genomes (KEGG), coding/noncoding co-expression (CNC), and gene ontology (GO) analyses revealed that differentially expressed mRNAs were associated with T-cell type selection, alpha-beta T cell activation, and the IFN-cellular response. KEGG identified a number of mRNAs as being involved in the T-cell receptor signaling pathway and the Jak-STAT signaling circuit [135]. Thus, it has been demonstrated that variable long ncRNA expression impairs T-cell and T helper cell activation, resulting in immunological deficits in tuberculosis patients [136]. Furthermore, microarray analysis revealed a greater role for non-coding RNAs in T cell regulation and related signaling networks. Furthermore, this aberrant expression may have an effect on the cAMP and calcium signaling pathways, as well as natural killer cell signaling pathways [137,138]. Differential expression of competitive endogenous RNAs (ceRNAs) was observed in ENST00000422183, ENST00000570366, NR 003142, and NR 038221 and was significantly linked with tuberculosis, implying that it may influence tuberculosis pathogenesis via its effect on the transduction of signals between immune cells and intracellular signaling pathways [139]. Ultimately, this analysis asserted that long ncRNAs serve as early detection biomarkers for tuberculosis. Another study in China examined 467 tuberculosis patients and 473 healthy controls, and lnc-AC145676 and lnc-TGS1–1 were analyzed. As determined by RT-qPCR analysis, both of these lncRNAs were significantly downregulated in tuberculosis patients. This could imply the involvement of lnc-AC145676. While, lnc-TGS1–1 significantly reduced infection risk with Mtb [140]. MiR-143, a miRNA that binds to lnc-TGS1–1, was found to be significantly upregulated during tuberculosis infection, resulting in decreased immune function via downstream gene downregulation. This could be because the expression of lnc-TGS1–1 had decreased, removing its sponge effect on miR-143. Furthermore, the absence of lnc-AC145676 increased miR-29a expression and interfered with the TLR signaling pathway and other immune response interactions in tuberculosis. Additionally, this study established a link between reduced lnc- TGS1–1 expression and the prevalence of thrombocytopenia in tuberculosis patients receiving anti-TB medication [141]. It was discovered that lnc-TGS1–1 and rs4737420 (a variant of lnc-TGS1–1) can function as prognostic markers for adverse drug reactions associated with anti-TB drugs (ATD-ADRs). Another study discovered that the patterns of RNA expression differ between patients with MDR-TB and drug-sensitive tuberculosis (DS-TB), as well as between the PBMCs of healthy people [142]. The majority of signaling pathways involved in the dysregulation of these lncRNAs have been identified, including those involved in Hippo signaling, NK cell-mediated cytotoxicity, and antigen presentation. Additionally, recently, single nucleotide polymorphisms (SNPs) in the lncRNA AC079767 were identified. Four genes have been linked with the growth of tuberculosis ADRs, and polymorphisms such as rs12477677 and rs1055229, as well as variants of the lncRNA AC079767, and may contribute to tuberculosis clinical presentation and may be potential biomarkers for the diagnosis and progression of tuberculosis [143]. Similarly, an analysis discovered that 2116 lncRNAs were differentially expressed in the plasma of tuberculosis infected persons, with 1102 lncRNAs being upregulated and 1014 being downregulated. The expression levels of ENST00000354432 and ENST00000427151 were confirmed in additional TB samples. It was proposed that the levels of lncRNA expression, particularly those of these two genuine long ncRNAs in plasma, could be used as potential biomarkers for early tuberculosis diagnosis [144].

**Table 1:** List of noncoding RNAs used in the etiology, diagnosis, and treatment of Tuberculosis.

Noncoding RNA	Function in Tuberculosis	Action	References
miR-26-5p	Etiology	Inhibition of innate immunity	[145]
miR-132-3p			[146]
miR-155-5p			[147]
miR-29-3p			[148]
miR-21-5p		Suppression of inflammation	[149]
miR-27b-3p			[150]
miR-99b-5p			[151]
miR-125-5p			[152]
miR-146a-5p			[153]
miR-223-3p			[154]
Let-7f			[155]
miR-20b-5p			[156]
miR-142-3p			[157]
miR-33		Inhibition of phagosome maturation and autophagy	[158]
miR-27a-5p			[159]
miR-125a-3p			[160]
miR-144-5p			[161]
miR-889-5p			[162]
miR-155-5p		Apoptosis Inhibition	[163]
miR-582-5p			[20]
miR-769-5p	Diagnosis	Downregulation in TB patients	[164]
miR-320a			[165]
miR-22-3p			[166]
hsa_circ_0001380			[167]
miR-423-5p		Upregulation in TB patients	[168]
miR-17-5p			[169]
miR-20b-5p			[170]
lncRNA LOC152742			[171]
hsa_circRNA_001937			[172]
has_circRNA_051239			[173]
hsa_circRNA_404022			[174]
has_circRNA_029965			[175]
lncRNAs NEAT1	Treatment	Downregulation during drug treatment, linked with disease improvement	[176]
lncRNAs NEAT2			[177]
lncRNA 152742			[178]
circTRAPPC6B			[179]
lncRNAENST00000429730.1		Downregulation during drug treatment, linked with entire inactivation of TB lesions from sputum negative patients	[180]
lncRNA MSTRG.93125.4			[181]

### 4.3. CircRNAs as a Diagnostic and Therapeutic Biomarker for Tuberculosis

Delay in diagnosis and treatment of tuberculosis is a serious threat to public health. However, the sensitivity of the tuberculosis diagnostic methods presently available is limited [182]. As a result, novel biomarkers are now required to assist in the early detection and treatment of TB in the lungs. Zhang et al. identified and synthesized ceRNAs for 170 dysregulated circRNAs in lungs infected with tuberculosis [183]. Their findings indicated that circRNA-linked ceRNA-mediated regulation of gene is critical for pulmonary tuberculosis pathogenesis. Huang et al. determined that has-circ-001937 has a substantial diagnostic value for tuberculosis (under curve area = 0.873). It was associated with the severity of tuberculosis and served as a TB-specific signature circRNA, with notably higher levels in TB-infected people compared to pneumonia and lung cancer [184]. Zhuang et al. discovered that hsa-circ-0005836 and hsa-circ-0009128 were considerably downregulated in the PBMCs of patients with active TB and healthy people [185]. The key roles of differentially expressed (DE) circular RNAs were found to be associated with immune system activation, implying a connection between tuberculosis infection and immune system activity. Qian et al. identified DE circRNAs in tuberculosis patients’ PBMCs and used seven of them to create an individual tuberculosis index [186]. Tuberculosis patients and healthy controls had a significantly higher tuberculosis index (under curve area = 0.946) in validated groups. Additionally, Yi et al. found the plasma circular RNA expression profiles of patients with active tuberculosis and discovered that patients with tuberculosis had significantly lower levels of hsa-circRNA-103571 expression [187]. Additional computational biology analysis demonstrated that hsa-circRNA-103571 regulated T and B-cell receptor signaling pathways. Numerous circRNAs have been identified as diagnostic markers for the infection of tuberculosis and future investigations might focus on nontuberculous mycobacteria (NTB), which shares clinical symptoms with tuberculosis, resulting in frequent misdiagnosis and mistreatment [188,189]. Furthermore, the majority of NTBs are highly resistant to anti-tuberculosis medicines, posing a significant infection burden on society [190]. Circulating RNAs may one day serve as a novel biomarker to aid physicians in differentiating tuberculosis infection from non-tuberculosis infections.

## 5. Methods for Profiling ncRNAs Expression

**Figure 4:**
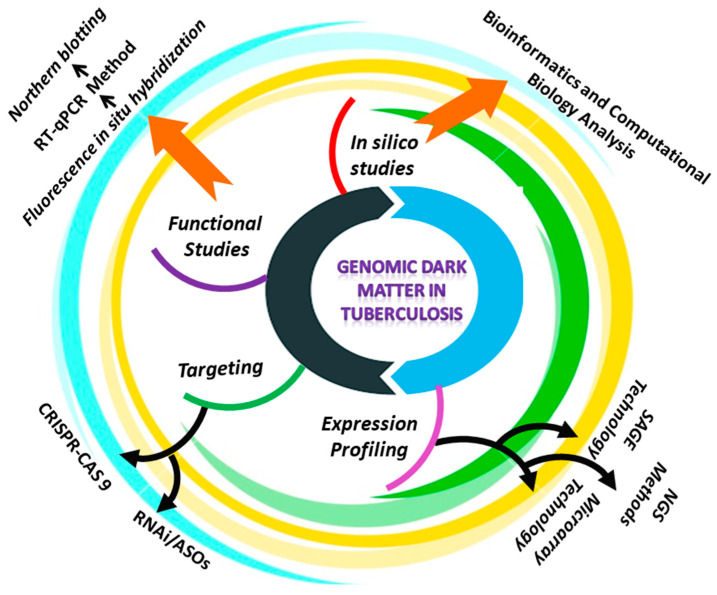
Schematic representation of the approaches reviewed in this study to investigate the role of ncRNAs in tuberculosis.

A common feature of tuberculosis in humans is aberrant ncRNA expression. Due to the specificity of ncRNAs for cell type, and tissue, RNA profiling has become a method for identifying valuable biomarkers of TB diagnosis, and metastasis [191]. When using both arrays and next generation sequencing (NGS) approaches for detecting and quantifying non-coding RNAs, several factors must be considered. In general, microRNAs and long noncoding RNAs are expressed at a lower level than messenger RNAs [192]. MiRNA profiling, necessitates the isolation of RNA to preserve the small fraction of RNA. Additionally, because microRNAs lack a conserved sequence, such as the poly (A) tail found in mRNAs, this class of non-coding RNAs must be detected selectively among various RNA species. Additionally, due to post-transcriptional modifications, miRNAs from the similar family can exhibit a high degree of divergence from the reference sequence [193]. When compared to mRNAs, lncRNAs share several characteristics with them, including size, transcription by RNA polymerase II, 5′-capping, and RNA splicing. Additionally, approximately 60% of the long ncRNAs contain a polyA tail. As a result, long ncRNAs may be profiled similarly to mRNAs, whereas microRNAs require a different strategy [194]. Despite this, the development of probes for the majority of lncRNAs is difficult to identify, because they are found at antisense transcripts of well-characterized protein-coding genes or intergenic regions with a high G-C content [195]. We describe several methods for comparing gene expression patterns in normal and TB-infected cells, in order to identify the noncoding RNAs that may be involved in tuberculosis (Figure 4).

### 5.1. Microarray

Despite their origins in the profiling of protein-coding mRNAs, microarrays are a well-known technique for profiling miRNAs and long noncoding RNAs [196]. This technique is based on the hybridization of labelled RNA targets to their precise and complementary probes using nucleic acids. Microarrays have a number of advantages, including high parallelism, being inexpensive, and the power to identify extremely low abundance of RNA, without using PCR enrichment [197]. Numerous miRNA profiling platforms make use of a variety of non-amplification-based direct miRNA labelling techniques. Along with mRNAs, lncRNA microarray platforms facilitate the systematic profiling of lncRNAs. In general, lncRNA platforms employ in vitro transcription (IVT) for amplification and are slightly more technically sophisticated than miRNA platforms, primarily in terms of the number of long ncRNAs analyzed [198]. For instance, the Arraystar Long ncRNA microarray profiles 39,317 lncRNAs; and Thermo Fisher Scientific’s Clariom D human array profiles over 55,900 human long ncRNA NONCODE transcripts. Current techniques for non-coding RNA analysis have many limitations, including a limited linear quantification range, probe design constraints on known sequences, the requirement for continuous annotation updates, and a relative quantification that is restricted to comparing different grades (for example, control versus infected). Yang et al. profiled 31 TB-patients using an Arraystar Human LncRNA Microarray technology and identified two long ncRNAs (MIR3945HG V1 and MIR3945HG V2) as new candidate markers for the diagnosis of tuberculosis [199]. A Tiling array is another microarray-based technique distinguished by the use of probes that span distinct chromosomal sequences, or the entire genome. Bertone et al. discovered 10,595 previously unknown transcribed sequences in 2004, but this technology has been largely superseded in modern biology by next-generation sequencing approaches [200,201].

### 5.2. Serial Analysis of Gene Expression 

SAGE is a breakthrough sequencing technique, designed specifically for the purpose of characterizing and quantifying the transcriptome, as well as noncoding RNAs [202]. It is based on the synthesis of small segments of unbiased cDNA sequences using restriction enzymes, followed by concatenation, cloning, and sequencing [203]. This technique has been employed in the “SuperSAGE” variant, which enables profiling of 26-basepair tags and in-tag-to-gene annotation at a lower cost/quality ratio. Gibb et al. used 24 million SAGE tags to determine the expression profile of long ncRNAs in 26 control and 19 tumor tissues [204].

### 5.3. RNA Sequencing (RNA-seq)

RNA-seq enables the identification and the quantification of all noncoding RNA groups by creating distinct cDNA libraries for each noncoding RNA type [205]. The desired transcripts are sequenced in massive parallel following the preparation of the cDNA library. While small RNA sequencing is appropriate for small noncoding RNAs, entire RNA sequencing is recommended for long noncoding RNAs, as many long noncoding RNAs are not poly-adenylated [206]. RNA-seq covers entire transcriptomes more thoroughly than a microarray. Notably, RNA-seq is a non-design-based technique that enables the finding of unknown/novel transcripts, as well as sequences with the difference of a single nucleotide, such as mutant or isoform-containing transcripts [207]. The primary drawbacks of RNA-seq are the complexity of the data analysis, and large number of deep reads needed to identify a small amount of target. Arnvig et al. published the first *M. tuberculosis* transcriptome investigation utilizing RNA-seq, examining whole-transcriptome expression throughout exponential growth and stationary phase [208]. Silva et al. used RNA-seq technology and identified three differentially expressed miRNAs (hsa-miR-486-3p, hsa-let-7g-5p, and hsa-miR-4732-5p) by differential expression analysis between tuberculosis patients and controls [209]. Luo et al. used RNA-seq technology to identified circRNA (hsa_circ_0001380) in peripheral blood, which can act as a biomarker for the diagnosis of tuberculosis [210]. Single cell transcriptomic sequencing is the cutting-edge application of RNA-seq. For instance, the designed primer-based RNA-seq approach (DP-seq) permits RNA amplification from 50 pg of sample, whereas Quartz-Seq is a single cell RNA-seq technique capable of revealing genetic differences amongst single cells of the same cell type, and cell cycle phase [211].

### 5.4. In Silico Investigation

Bioinformatics expertise is required to analyze the data generated by a variety of platforms, including those described above for microarray and NGS technologies [212]. NGS is increasingly being employed to decipher the genetic underpinnings of intra- and inter-individual variability, which is becoming more critical in the personalized medicine era [213]. The analysis of ncRNA can be used to accomplish a variety of objectives, including the detection and annotation of new non-coding, expression pattern profiling, authentication, and reconstruction of previously identified non-coding, as well as integrative analysis of their behavior and functions [214]. When dealing with array data, bioinformatics analysis entails the following steps: (i) identifying genes that are expressed differently between two groups (control versus TB-infected samples, pharmacologically treated vs. untreated cells); (ii) clustering, which classifies genes according to their level of expression; (iii) classification; and (iv) pathway and interaction network analysis. The processing of microarray raw data is divided into four stages: (1) pre-processing, which involves background adjustment, normalization, and summarization; (2) annotating pre-processed data to enrich it; and (3) data mining and statistical analysis [215]. For preprocessing microarray data, the algorithms GCRMA, RMA, MAS4.0, and PLIER are all well-known [216]. Background correction is required to eliminate the noise generated by non-specific hybridization in the optical detection system. Normalization is required within and between arrays, to eliminate the systemic technical artefacts caused by variations in reverse transcription, labelling, or hybridization efficiencies. Summarization is a technique that combines signals from multiple probes directed at the same transcript and distributed across the array [217]. Once the data has been summarized, it can be annotated with additional info such as gene symbols or functions. Data mining is a technique that involves comparing the sets of samples to determine which genes are expressed differentially, based on their expression values [218]. Numerous methods for visualizing and analyzing gene expression data are applicable to microarray and RNA-seq experiments, including gene set enrichment analysis, pathway analysis (WikiPathway, Reactome, KEGG, and Ingenuity), clustering analysis, and network analysis [219].

In RNA-seq technology, ncRNAs analysis begins with the un-processed NGS data. The first step is to eliminate low-quality reads from the unprocessed data. This is typically accomplished using raw files having FASTA-FASTQ-encoded short reads with tools that map the sequences to reference data. Blat, SHRiMP, MAQ, LastZ, and FASTX-Toolkit are just a few examples of such programs. The transcripts are assembled using BowTie or TopHat, following filtering. Following assembly, tools such as Bowtie are used to filter known DNA sequences. Following this phase, all DNA sequences that may denote ncRNAs must be evaluated and mapped using tools such as CPAT or Pfamscan [220]. This step is recommended with use of the NCBI NT and NR databases, because they contain sequences from all species. Sun et al. described lncRNAscan, a pipeline for identifying new lncRNAs from transcripts sequenced by RNA sequencing [216]. The pipeline explained above has been utilized in almost every bioinformatics study on noncoding RNAs that has corroborated the pattern of ncRNA expression with the clinical outcome of diseased-persons. Ultimately, the role of ncRNAs must be deduced through analysis of existing databases that contain a lot of ncRNA sequences and, when available, data from biological reports (Table 2) [221]. While these databases can be queried to find known noncoding RNAs within a given set of data, many known noncoding RNAs are valid only for well-characterized species, due to a lack of conservation. Jonathan et al. used the sRNAPredict2 program, applied for the identification of ncRNAs in the *M. tuberculosis* genome [222]. Reddy et al. published an integrated database named “The Tuberculosis Database (TBDB)”, which provides access to tuberculosis genetic resources, critical for the discovery and development of tuberculosis medicines, vaccines, and diagnostic biomarkers [223].

## 6. Methods for Validating ncRNA Expression

It is necessary to validate microarray/RNA-seq data, and in-silico predictions. Noncoding RNAs can be quantified using a variety of techniques, including northern blot (NB), reverse transcription quantitative polymerase chain reaction (RT-qPCR), and fluorescence in situ hybridization (FISH) [243,244].

### 6.1. Northern Blotting (NB)

NB was the first method used to analyze the splicing variants of certain non-coding RNA’s gene expression [245]. The procedure begins with gel electrophoresis separation of RNA samples, then RNA transfer to nylon membrane, hybridization with an RNA probe, and lastly detection with an RNA probe. Wang et al. validated 28 novel small RNA-encoding regions in *M. tuberculosis* by northern blotting, which were identified from RNA-seq data [85]. Their study will help in the future to elucidate the complexity of gene expression regulation, mediated by sRNA in *M. tuberculosis* [246].

The primary disadvantage of this technique is its lower sensitivity, and prolonged execution time. Samples require a good amount of total RNA, which is extremely difficult to obtain when miRNAs are scarce or cell or tissue RNA sources are limited [247]. Additionally, the classical protocol’s use of isotope labelling is risky, and as a result, a large number of institutions prohibit its use. Numerous improvements to the classical method have been made in recent years, with the use of non-radioactive labelling agents, such as modified digoxigenin (DIG) probes [248].

### 6.2. RT-qPCR

RT-qPCR is a commonly used method for the identification and quantification of noncoding RNAs, due to its ease of integration into laboratory workflows [249]. As demonstrated by He et al. in their analysis of the differentially expressed profile of miRNAs in lung cancer patients’ peripheral blood, this technique is frequently used to validate microarray data. TaqMan and SYBR green assays are used in the RT-qPCR technique. The reverse transcription step is ncRNA-specific [250]. The TaqMan assay reverse transcripts miRNAs using a particular stem loop reverse transcription primer, whereas the SYBR green assay modifies a miRNA sequence by adding a poly-A tail, to facilitate primer binding. In the case of lncRNA, reverse transcription using random or particular primers is followed by qPCR employing TaqMan or SYBR green chemistry, to monitor the reaction product buildup in real time. Zhao et al. discovered the serum lncRNA n335659, which could be used as a biomarker for tuberculosis, and validated this biomarker using RT-qPCR technology [251].

### 6.3. FISH

Recent advancements in probe technology and identification methodologies have increased the imaging of ncRNAs via the FISH method, which is based on the employment of fluorescent probes with a high degree of complementarity to the nucleic acid sequence [252,253]. FISH provides novel information about the biological function of ncRNAs, by determining their spatial–temporal expression and subcellular localization. Confocal microscopy for FISH, for example, revealed that the long noncoding RNA NKILA is required in the cytoplasm, because it inhibits NF-B activation by stabilizing the NF-B/I-B complex; thereby, inhibiting cancer-associated inflammation; while, because of the small length and presence of repetitive sequences, using fluorophore-labeled DNA/RNA probes is extremely difficult. Branched-DNA probes, LNA probes, multiple oligo probe sets, are all examples of multiple oligo probe sets that have been fluorophore labeled [254]. In particular, it has been demonstrated that the usage of hapten-labeled LNA oligos is extremely advantageous for miRNA detection in clinical tissue samples, while there are few reports on lncRNA detection [255]. To circumvent these methods’ limitations in detecting low-abundance non-coding RNAs, scientists have established and applied single-molecule RNA FISH, a technique based on the hybridization of several small fluorescently-labelled oligonucleotides from a single cell. The risk of off-target hybridization is minimized by employing a single well-designed oligo probe, with low cross-reactivity to other RNAs [256].

## 7. Potentially Beneficial Therapeutic Interventions

The dysregulation of ncRNAs in TB-infected cells, in terms of expression profile and interactome, provides a rationale for considering them as a group of possible therapeutic markers [257]. Knowing the complexity of their promising mechanism of action, a variety of genomic and practical techniques have been designed to target ncRNAs directly/indirectly. We discuss the following: (1) post-transcriptional RNA degradation using synthetic antisense oligonucleotides (ASOs); (2) modulation of non-coding RNA genes via genome editing technologies. We discuss various preclinical studies that demonstrated the effectiveness of these strategies in functional investigations. Despite the fact that all of these approaches have the potential to be effective therapeutic interventions, numerous constraints, such as delivery systems, must be addressed before they can be translated into clinical practice.

### 7.1. Noncoding RNAs Targeting: ASOs

ASOs are sequences of synthetic nucleic acids which bind to complementary RNA substrates via Watson–Crick base pairing [258]. ASOs have been engineered to maintain drug-like characteristics through chemical variation of endogenous nucleotides. Phosphoro-thioate alteration of the linker protects ASOs against nuclease degradation and prolongs their half-life in blood, while maintaining RNaseH activity. These first-generation ASOs, which contained only deoxy residues, had a short clinical life. ASOs of the second generation have a core area of around ten phosphoro-thioate nucleotides bordered by nucleotides changed at the sugar (“gapmer” design). Third-generation ASOs are constructed of LNA-modified antisense oligonucleotides gapmers, with LNA-enriched flanking regions and a DNA-free middle gap [259,260]. LNAs are analogues of nucleotides, where the ribose ring is locked between the 2′ oxygen and the 4′ carbon through a methylene bridge. Gcanga et al. identified lincRNA-MIR99AHG, which is upregulated in macrophages but downregulated after Mtb infection and in patients with active tuberculosis [261]. However, they indicated for the first time that treatment with MIR99AHG ASOs significantly reduced the pathogenesis of Mtb infection.

### 7.2. Non-Coding RNAs Targeting: RNAi

RNA interference (RNAi) is an endogenous process of post-transcriptional regulation, which functions by pairing endogenous or exogenous double stranded RNA with a target mRNA [262]. To be precise, a dsRNA is first split into a 21-nucleotide RNA sequence known as siRNA by Dicer, and subsequently put into the cytosolic RISC. The promoter strand is removed, the guide strand is linked with the target mRNA, and silencing is produced by degradation, depending on complementarity. This physiological process has been extensively used experimentally in molecular oncology, for therapeutic purposes, and subsequently improved to execute high-throughput screening, employing the siRNAs’ pool [263]. Indeed, many libraries targeting microRNAs and long noncoding RNAs have been created, resulting in the discovery of non-coding RNAs involved in drug response. For instance, utilization of genome-wide microRNA libraries enabled the detection of miR 195’s synergic involvement in the response of microtubule-targeting agents to lung cancer [264]. On other hand, synthetic siRNAs have been employed to induce ncRNA degradation in a therapeutic setting. Additionally, siRNA-mediated inhibition of MALAT1 revealed that it plays a significant role in temozolomide resistance in glioblastoma multiforme, as its inhibition repaired drug sensitivity, while diminishing cancer stem cell stemness and proliferation [265].

### 7.3. ncRNAs Editing with CRISPR-Cas9

Clustered regularly interspaced short palindromic repeats (CRISPR)-associated nuclease 9 (CRISPR/Cas9) is a genome editing technique that functions like a molecular “scissor”. It was developed by manipulating the adaptive immune system of prokaryotes, so as to induce a distinct genetic alteration in eukaryotes using guide RNA (gRNA) and Cas9 protein [266]. gRNA has a length of 20-nt, and is homologous to a portion of the targeted DNA flanked by a three bp protospacer adjacent motif (PAM) sequence recognized by Cas9, an endonuclease capable of inducing a double stranded break (DSB). Cas9-mediated DSBs can repair using non-homologous end joining (NHEJ), by generating tiny indels disrupting the targeted locus (knock-out (KO) method) or via homology directed repair (HDR), in which donor DNA is used to insert a required sequence (knock-in). Numerous validation findings have been conducted using this technique, to determine the role of selected long noncoding RNAs [267,268]. For instance, CRISPR-Cas9 technology has been used to establish the functions of (1) LncRoR as an activator of the MAPK/ERK pathway, and (2) LncAK023948 as a positive regulator of the Akt pathway. By targeting the miRNA biogenesis region, the CRISPR-Cas9 method has also been utilized to significantly lower miRNA expression in vitro and in vivo. Diverse applications of the CRISPR-Cas9 method have enabled the identification of miR-210’s onco-suppressive involvement in renal cell cancer cell lines and miRNA182-onco-suppressive 5p’s activity in chronic myeloid leukemia [269]. Additionally, several research studies examined prospective delivery routes for miRNA therapies using the CRISPR-Cas9 system.

Given the enormous influence of this technique on molecular investigations, CRISPR-Cas9 has much improved the induction of progressively precise genetic modifications, up to the level of base editing [270]. Liu et al. conducted a illustrative investigation of CRISPR-interference-based screening in seven transfected cell lines, using 1600 lncRNAs as targets [271]. They discovered 499 lncRNA loci essential in cellular development and tissue-specific transcriptional control. Kurata et al. found miRNAs linked with cell fitness using a pooled CRISPR-Cas9 library based on 1600 annotated human miRNA stem-loops. For the first time, Liu et al. artificially synthesized a circRNA that was capable of inhibiting the proliferation of stomach cancer cells by miR-21 sponging [272]. This report generated a novel concept for the future clinical application of circular RNAs in tuberculosis. Additionally, disease-promoting circRNAs could be targeted using CRISPR/Cas9-based gene editing, to specifically target the back-splice junction of circRNAs [273].

The primary constraint on this technology is the off-target impacts, despite the fact that numerous measures are taken to address or at least mitigate this critical issue. In terms of clinical translation, CRISPR-Cas9-based techniques are still in their infancy, owing to the possibility of an unfavorable immune response to bacterial Cas9, which is often delivered via viral vectors, and due to ethical problems inherent in human genome editing applications [274].

## 8. Conclusions

In the current review, the research approaches used to explore the function of miRNAs, lncRNAs, and circular RNAs in tuberculosis were detailed. Progression of tuberculosis disease could be stopped by decreasing or increasing the expression of particular noncoding RNAs and then blocking/activating the genes they regulate. Indeed, they can have therapeutic potential too. The availability of new strong sequencing and molecular tools has enabled the overcoming of various constraints, including ncRNAs’ low quantity, subcellular spatial localization, and their instability. In terms of identification and functional validation, advances in wet laboratory techniques, combined with in-silico tools, have dramatically increased our understanding of the genome’s dark matter. Further developments in the coming years are likely to improve our understanding of the regulatory network behind ncRNA perturbations and, more importantly, facilitate the translation of experimental findings from bench to bedside. Given the existing data, translational research to test and include all examined markers into point-of-care tuberculosis testing is a worthwhile endeavor. This will enable research focused at overcoming the diagnostic problems associated with tuberculosis illness.

## Figures and Tables

**Table 2:** List of bioinformatics resources used in the prediction and analysis of ncRNAs.

Tool Name	Description	References
miRanalyzer	miRNA detection tool for NGS experiments	[224]
miRTools	Toolbox for miRNA discovery and profiling	[225]
miRiadne	Tool for integrating the miRNA nomenclature	[226]
miRBase	Database of miRNA sequence and annotation	[227]
DIANA-mirGen	A tool to index promoters and regulator for miRNA	[228]
miRStart	miRNA’s Transcription Start Sites database	[229]
miRWalk 2.0	Tool to predict miRNA-target interaction using Artificial intelligence algorithm	[229]
MatureBayes	miRNA-target prediction tool	[230]
miRanda	Tool to predict miRNA target using free energy feature	[231]
LNCipedia	lncRNA database	[232]
LNCBook	lncRNA database	[233]
LncDisease	lncRNA-disease associations predicting tool	[234]
NONCODE	Integrated knowledge database for ncRNA research	[235]
StarBase v2.0	Tool to predict miRNA-ceRNA interaction	[236]
NRED	Database for expression information of lncRNAs	[237]
Circ2Traits	Toolbox for circularRNA discovery and analysis	[238]
CircRNABase	Tool to predict miRNA-circRNA interaction	[239]
circBase	circularRNA database	[240]
Circtools	Toolbox for circularRNA research	[241]
CircMarker	Tool for circularRNA detection	[242]

## Data Availability

The data presented in this study are available within the article.
